# Deciphering the molecular basis of arsenic/interferon-alpha induced eradication of leukemia initiating cells in adult T cell leukemia

**DOI:** 10.1186/1742-4690-12-S1-P2

**Published:** 2015-08-28

**Authors:** Hiba El Hajj, Nadim Tawil, Rita Hleihel, Mark Jabbour, Ghazi Zaatari, Zafar Nawaz, Maysaloun Merhi, Edna Wang, Hideki Hasegawa, William Hall, Olivier Hermine, Hugues De Thé, Ali Bazarbachi

**Affiliations:** 1 American University of Beirut, Beirut, Lebanon; 2 Hamad medical corporation, Qatar; 3 Sidra Medical and Research Center, Qatar; 4 National Institute of Infectious Diseases, Tokyo, Japan; 5 University College Dublin, Dublin, Ireland; 6 Necker Hospital, Paris, France; 7 Hopital Saint Louis, Paris, France

## 

Adult T-cell leukemia (ATL) is a severe, chemotherapy-resistant malignancy caused by chronic HTLV-I infection and associated with dismal long-term prognosis. The HTLV-I oncoprotein Tax initiates ATL in transgenic mice. We have previously shown that arsenic trioxide and interferon-α (IFN) combination, known to trigger proteasome mediated Tax proteolysis and apoptosis in HTLV-I transformed or fresh ATL cells, cures Tax-driven ATL in mice. Unexpectedly, this combination therapy of primary donor mice abrogated leukemia initiating cells (LIC) engraftment into untreated secondary recipients, whereas the primary tumor bulk still grew in the primary hosts, only to ultimately abate later on. This loss of initial transplantability required proteasomal function. Furthermore, these preclinical results led to very promising improvement in ATL patients when arsenic was added to zidovudine and IFN. To decipher the molecular basis of LIC eradication in the murine ATL Tax transgenic model, primary ATL donor mice were treated with arsenic/IFN for 3 days starting on day 18 post-leukemic cells inoculation and sacrificed at day 21. Spleen derived ATL cells were intraperitoneally injected into secondary recipient SCID mice that were left untreated and were sacrificed on a weekly basis. Whereas, secondary mice derived from untreated primary succumbed from ATL between 3 and 4 weeks after transplantation, we observed an unprecedented biphasic growth in secondary mice transplanted from arsenic/interferon treated primary mice. Indeed, the tumor increased progressively until week 5 with organ infiltration and leukocytosis, followed by a spontaneous and dramatic tumor regression for 2 weeks before a second phase of tumor growth that culminates in death from ATL at around 9-10 weeks. ATL cells derived from the initial phase of growth (week 4) were healthy, TUNEL negative and showed a normal capacity in inducing ATL in tertiary hosts. Strikingly, ATL cells derived from the second phase of growth (weeks 7 to 9) showed a progressive increase in p53 expression and TUNEL positivity reaching more than 50% at week 9, and lost their capacity to induce ATL in tertiary hosts. Finally, gene expression profile revealed an arsenic/interferon signature that was similar at Week 4 and Week 8 as well as a specific signature at Week 8 concomitant with the loss of LIC activity. These results represent a unique and novel model of studying treatment-induced LIC eradication.

